# Differential Distribution of Major Brain Gangliosides in the Adult Mouse Central Nervous System

**DOI:** 10.1371/journal.pone.0075720

**Published:** 2013-09-30

**Authors:** Katarina Vajn, Barbara Viljetić, Ivan Večeslav Degmečić, Ronald L. Schnaar, Marija Heffer

**Affiliations:** 1 Department of Medical Biology, University of Osijek School of Medicine, Osijek, Croatia; 2 Department of Chemistry, Biochemistry and Clinical Chemistry, University of Osijek School of Medicine, Osijek, Croatia; 3 Animal Facility, University of Osijek School of Medicine, Osijek, Croatia; 4 Departments of Pharmacology and Neuroscience, The Johns Hopkins School of Medicine, Baltimore, Maryland, United States of America; University of Missouri, United States of America

## Abstract

Gangliosides - sialic acid-bearing glycolipids - are major cell surface determinants on neurons and axons. The same four closely related structures, GM1, GD1a, GD1b and GT1b, comprise the majority of total brain gangliosides in mammals and birds. Gangliosides regulate the activities of proteins in the membranes in which they reside, and also act as cell-cell recognition receptors. Understanding the functions of major brain gangliosides requires knowledge of their tissue distribution, which has been accomplished in the past using biochemical and immunohistochemical methods. Armed with new knowledge about the stability and accessibility of gangliosides in tissues and new IgG-class specific monoclonal antibodies, we investigated the detailed tissue distribution of gangliosides in the adult mouse brain. Gangliosides GD1b and GT1b are widely expressed in gray and white matter. In contrast, GM1 is predominately found in white matter and GD1a is specifically expressed in certain brain nuclei/tracts. These findings are considered in relationship to the hypothesis that gangliosides GD1a and GT1b act as receptors for an important axon-myelin recognition protein, myelin-associated glycoprotein (MAG). Mediating axon-myelin interactions is but one potential function of the major brain gangliosides, and more detailed knowledge of their distribution may help direct future functional studies.

## Introduction

**Figure 1:**
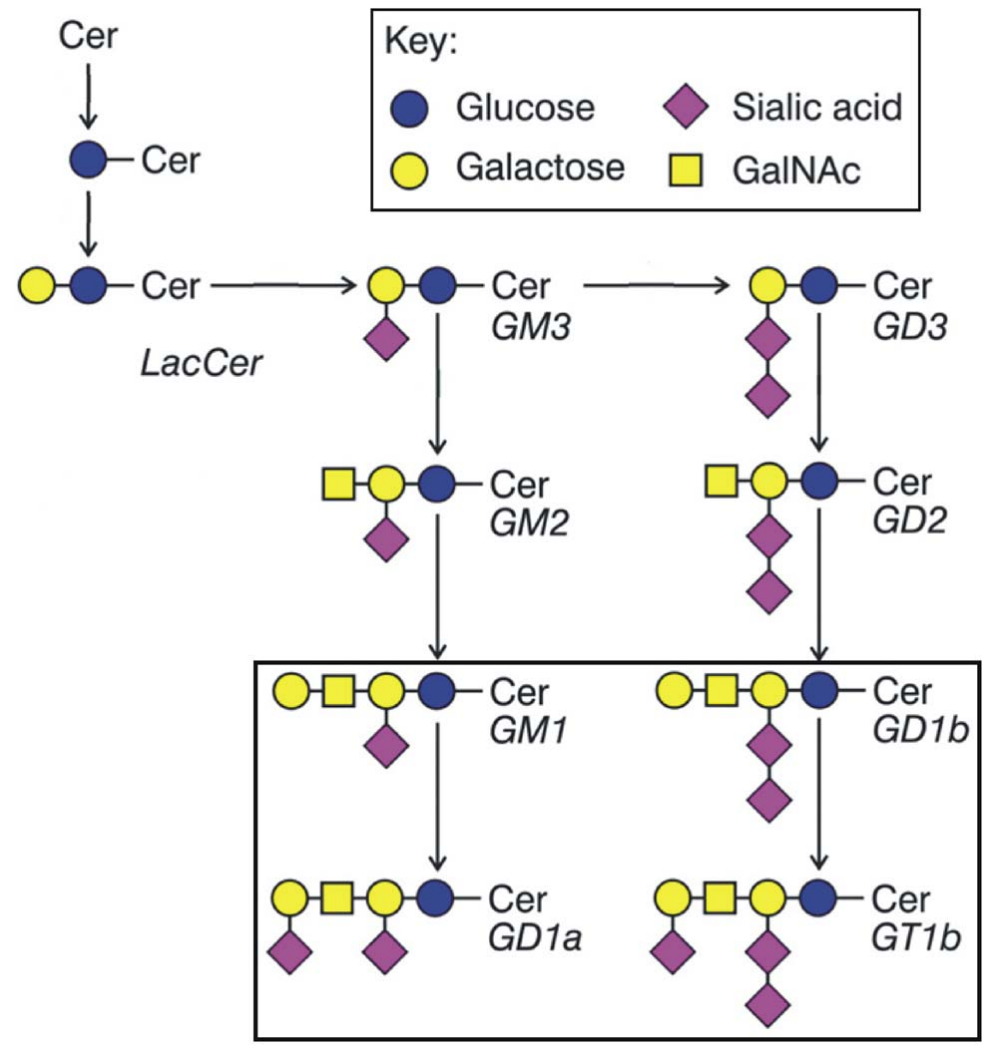
Structure and biosynthetic pathways of the major brain gangliosides [nomenclature is that of Svennerholm [40]]. Cer, ceramide; LacCer, lactosylceramide. GM1, GD1a, GD1b and GT1b comprise up to 97% of all gangliosides in the human central nervous system (boxed area).

Gangliosides, sialic acid-containing glycosphingolipids, are expressed widely in vertebrate tissues but at particularly high abundance in the brain, where they are major cell surface determinants on nerve cells. Four ganglioside structures, GM1, GD1a, GD1b and GT1b (Fig. 1) constitute 97% of all gangliosides in normal human brain, and the same four gangliosides similarly dominate brain ganglioside expression in mammals and birds [1]. Gangliosides are found primarily in the outer leaflet of plasma membranes where they are anchored via their ceramide lipid moiety, with their glycan structures extending into the extracellular space. They engage molecules laterally - in their own membranes - to regulate cell signaling, and they engage molecules on apposing cells to regulate cell-cell interactions. In combination, ganglioside recognition leads to altered cell signaling and changes in cell function and physiology [2]. Mice genetically engineered to lack major brain gangliosides appear to develop normally, but demonstrate progressive nervous system deficits, especially in axon-myelin interactions [3]. A rare human genetic disorder resulting in congenital loss of complex gangliosides is more severe, resulting in neuromuscular and cognitive developmental stagnation, blindness, and seizures [4].

Knowledge of the distribution of major gangliosides in the brain informs theories about their functions. The distributions of major brain gangliosides in rodent and human CNS have been studied using chemical and immunohistochemical methods [5]–[13]. More recently, imaging mass spectrometry (IMS) has revealed remarkably subtle molecular distributions of ganglioside sub-species [14]–[17]. However, the results sometimes conflict, possibly due to limitations in anti-ganglioside antibody specificities [6], [9], [10], tissue fixation methods that disrupt gangliosides [6], [8]–[10], ganglioside degradation during analysis [14] or detergent-mediated redistribution in tissues [9], [12].

To reassess ganglioside distribution in the adult mouse CNS, we used highly specific IgG-class monoclonal antibodies (mAb) raised against each of the major brain gangliosides. Since mice fail to raise a robust IgG response to self-gangliosides, we successfully raised these mAb’s in mice genetically engineered to lack complex gangliosides (*B4galnt1*-null mice) [11], [18]. We used mild fixation of tissues (4% paraformaldehyde) under conditions that preserve gangliosides when compared to unfixed tissues [7], [19]. Finally, to avoid artifactual tissue redistribution we did not use detergents [20], [21]. The resulting differential distributions of the four major brain gangliosides have implications for understanding their functions.

## Materials and Methods

### Ethics, Animals and Tissue Collection

Fourteen brains and spinal cords were obtained from 3-month-old wild type (C57Bl/6 strain) female mice. The protocol was approved by Ethical Committee of University of Osijek School of Medicine and Johns Hopkins University Animal Care and Use Committee. Johns Hopkins is accredited by the Association for the Assessment and Accreditation of Laboratory Animal Care (AAALAC) International. The protocol also complied with Directive 2010/63/EU of the European Parliament and of the Council on the protection of animals used for scientific purposes.

Mice were deeply anesthetized with isoflurane and consequently transcardially perfused with phosphate buffered saline (PBS) and 4% paraformaldehyde (PFA) in PBS. The brains and spinal columns were dissected, additionally fixed in 4% PFA for 24 hours and cryoprotected in gradients of sucrose (10–30% w/v). After cryoprotection, spinal cords were dissected from the spinal column and the specimens (brains and spinal cords) snap frozen by immersion in cold 2-methylbutane.Tissues were cut on a cryostat and immunohistochemistry was performed using 20–35 µm thick free-floating sections.

### Primary Antibody Characterization

The primary antibodies used in this study are listed in Table S1. Highly specific IgG-class monoclonal antibodies against the four major CNS gangliosides were raised in *B4galnt1*-null mice that lack complex gangliosides and thus exhibit a robust IgG response to administered gangliosides [11], [18]. Each batch of antibody produced was tested with ELISA for the antibody specificity. No staining was present when anti-ganglioside antibodies were used on brain sections of *B4galnt1*-null mice that lack complex gangliosides.

Antibodies to myelin-associated glycoprotein (anti-MAG, Chemicon, Temecula, CA; MAB1567, clone 513) and myelin basic protein (anti-MBP, QED Bioscience, San Diego, CA) were used as markers of myelinated fibers. Anti - MAG antibody detects MAG in immunohistochemistry on frozen sections, immunocytochemistry and Western blot and is routinely evaluated by the manufacturer by staining of myelinated fibers of rat hippocampus (manufacturer’s technical sheet). In our hands, anti - MAG antibody stained all white matter tracts in mouse CNS at the concentration of 1.2 µg/ml. No staining was present when anti-MAG antibody was used to stain *Mag*-null mice.

Anti - MBP antibody reacts with residues 130–136 in human myelin basic protein (21.5 kD and 18.5 kD molecular forms) and it also recognizes primate, rabbit, sheep, goat, rat and mouse myelin basic protein (manufacturer’s technical sheet). In our hands, anti-MBP antibody was shown to stain all white matter tracts in mouse CNS at the concentration of 1.5 µg/ml.

For detection of brainstem catecholaminergic neurons we used an antibody against tyrosine hydroxylase (TH) (anti- tyrosine hydroxylase, AB152, Millipore, Billerica, MA) at a dilution of 1: 1000. Each batch of anti-TH antibody is routinely evaluated by the manufacturer by Western Blot on PC12 lysates where it labels a single band at approximately 62 kDa (reduced) corresponding to tyrosine hydroxylase (manufacturer’s technical sheet). In our hands, anti–TH antibody stained catecholaminergic neurons of medial and lateral parabrachial nuclei, locus coeruleus, caudal ventrolateral medulla and solitary nucleus.

### Immunohistochemistry

All steps of ganglioside immunohistochemistry were performed at 4°C and without the use of any detergent. The tissues were blocked in 1% bovine serum albumin (Sigma-Aldrich, St. Louis, MO), 5% goat serum (Invitrogen, Carlsbad, CA) in PBS. Immunohistochemistry of myelin-associated glycoprotein (MAG) and myelin basic protein (MBP) was performed using 1% Triton X-100 in the blocking solution.

After washing the free-floating tissues, primary antibody binding was detected with biotin-SP-AffiniPure goat anti-mouse IgG (H+L) (Jackson Immunoresearch Labs., West Grove, PA) at 1 µg/ml final concentration followed by Vector Elite peroxidase kit (Vector Laboratories, Burlingame, CA) and developed with SIGMA*FAST*™ DAB with Metal Enhancer (Sigma-Aldrich, St. Louis, MO). Stained sections were slide-mounted, air dried and scanned with Nikon Super CoolScan 9000 ED scanner prior to coverslipping with Vectamount (Vector Laboratories, Burlingame, CA). Additional images were captured using a Zeiss Axioskop 2 MOT microscope fitted with an Olympus D70 camera. Images were assembled in CorelDraw 12 software and, after assembly, were adjusted for contrast, intensity and brightness.

### Co-localization

Co-localization experiments were performed using affinity-purified rabbit anti- tyrosine hydroxylase (TH) antibody and monoclonal anti-ganglioside antibodies as previously described. Primary TH antibody binding was detected using biotinylated goat anti-rabbit IgG (H+L) (Jackson), and streptavidin Alexa Fluor® 488 conjugate (Invitrogen, Carlsbad, CA). Anti-ganglioside antibodies were detected using Alexa Fluor® 546 goat anti-mouse IgG (H+L) (Invitrogen).

### Anterograde Axon Labeling

To determine ganglioside expression in white matter tracts, five mice were subjected to anterograde labeling. Craniotomy was performed on anesthetized mice and sensorimotor cortex was injected with one injection of 0.2 µl 10% (w/v) 10,000 MW biotinylated dextran amine (BDA, Invitrogen). After 2 weeks mice were perfusion-fixed, brains and spinal cords were recovered as described above, cryosections prepared, and gangliosides detected using immunohistochemistry. BDA-labeled axons were detected with streptavidin Alexa Fluor® 488 conjugate, whereas anti-ganglioside antibodies were detected using Alexa Fluor® 546 goat anti-mouse IgG.

### Fluorescent Microscopy

Fluorescent immunohistochemical images were obtained using a Zeiss LSM 510 inverted confocal microscope (The Johns Hopkins School of Medicine Microscope Facility), assembled in CorelDraw 12 software and assembled images adjusted for contrast, intensity and brightness.

### Qualitative Analysis of Immunohistochemical Reactivity to Gangliosides GD1a and GT1b

Qualitative analysis of immunohistochemical reactivity to gangliosides GD1a and GT1b was performed on images taken with the same exposure times by two independent observers. The relative expression (+++, strong signal; ++, moderate signal; +, weak signal, −, no signal) of gangliosides GD1a and GT1b was compared to negative control on each mouse brain region listed in Table S2. Images of different brain regions were taken at different exposure times, so the comparisons were made between different antibodies in one brain region, but not between different brain regions.

## Results

### General Expression Patterns of Major Brain Gangliosides

**Figure 2:**
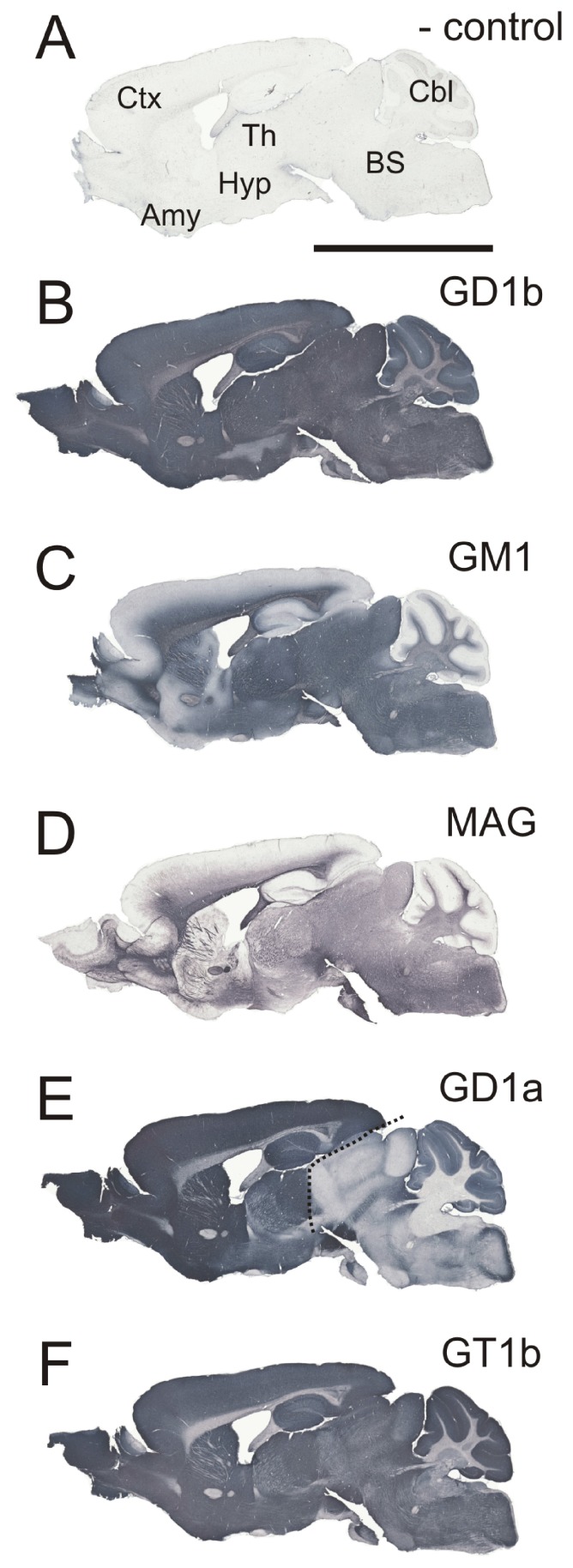
Differential distribution of gangliosides GD1b (B), GM1 (C), GD1a (E, the line demarcates the border from which the expression significantly decreases), GT1b (F) and MAG (D) in sagittal sections of adult C57Bl/6 mouse brain. The negative control (A) was performed by omitting the primary antibody. Scale bar: 5000 µm.

The expression patterns of gangliosides GM1, GD1a, GD1b and GT1b were studied using immunohistochemistry on adult C57Bl/6 mouse brains and spinal cords cut in three planes (coronal, sagittal and horizontal). GD1b expression is abundant in both gray and white matter throughout the mouse brain (Fig. 2B). In contrast, expression patterns of the other three major brain gangliosides appear to be enhanced in certain brain regions. GM1 is present throughout white matter (Fig. 2C), and generally follows the same expression pattern as MAG, an established myelin marker (Fig. 2D), although it is also found in some brain nuclei. Gangliosides GD1a and GT1b are predominately distributed in gray matter, but are also found in some white matter tracts (Figs. 2E and 2F, respectively). The distribution of GD1a and GT1b is similar in rostral parts of telencephalon, but, from the level of anterior pretectal nucleus and superior colliculus, the expression of GD1a is limited only to few structures in the brainstem (Fig. 2E, the line demarcates the brainstem border).

### Telencephalon

#### Olfactory bulb

**Figure 3:**
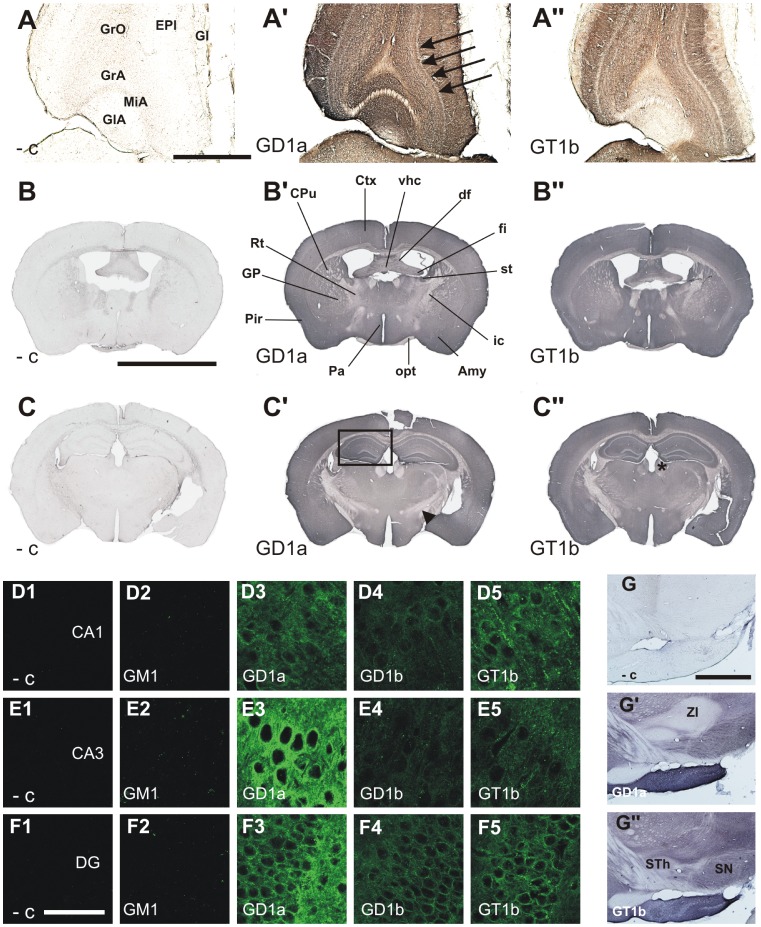
The expression of gangliosides GM1 (D2, E2, F2), GD1a (A’, B’ C’, D3, E3, F3, G’) and GT1b (A’’, B’’, C’’, D4, E4, F4, G’’) in mouse telencephalon and diencephalon. Horizontal sections of olfactory bulb (A, A’, A’’; mitral cell layer of olfactory bulb is pointed out with black arrows). Coronal sections of adult mouse brain at coordinates: interaural line 3.22 mm, bregma –0.58 mm (B, B’, B’’) and interaural line 2.10 mm, bregma –1.7 mm (C, C’, C’’). Hippocampal formation is boxed in C’, arrowhead in C’ shows zona incerta and asterix in C’’ indicates medial and lateral habenular nuclei. Higher magnifications of CA1 and CA3 fields of pyramidal cell layer of hippocampus (D1–D5 and E1–E5, respectively) and granule cell layer of dentate gyrus (F1–F5). Sagittal sections of zona incerta (ZI), subthalamic nucleus (STh) and substantia nigra (SN) (G-G’’). The negative controls were performed by omitting the primary antibody (A, B, C, D1, E1, F1, G). Amy, amygdala; CA1, CA1 field of pyramidal cell layer of hippocampus; CA3, CA3 field of pyramidal cell layer of hippocampus; CPu, caudate putamen (striatum); Ctx, cortex; df, dorsal fornix; DG, dentate gyrus; EPl, external plexiform layer of olfactory bulb; fi, fimbria of the hippocampus; Gl, glomerular layer of olfactory bulb; GlA, glomerular cell layer of accessory olfactory bulb; GP, globus pallidus; GrA, granule cell layer of accessory olfactory bulb; GrO, granule cell layer of olfactory bulb; ic, internal capsule; MiA, mitral cell layer of accessory olfactory bulb; opt, optic tract; Pa, paraventricular hypothalamic nucleus; Pir, piriform cortex; Rt, reticular nucleus (prethalamus); st, stria terminalis; vhc, ventral hippocampal commissure. Scale bars = 1000 µm in A-A’’, G-G’’; 4000 µm in B-C’’ and 50 µm in D1–F5.

GD1a shows strong immunoreactivity in all layers of olfactory bulb and accessory olfactory bulb (Fig. 3A’), including the mitral cell layer (Fig. 3A’, arrows), whereas GT1b is only weakly expressed in the glomerular layer of olfactory bulb and all layers of accessory olfactory bulb (Fig. 3A’’). GD1b is abundantly expressed in all layers of olfactory bulb and accessory olfactory bulb, whereas GM1 is limited to the intrabulbar part of the anterior commissure.

#### Cerebral cortex

Strong expression of gangliosides GD1a, GD1b and GT1b is found in all fields and layers of cerebral cortex (Figs. 2B, E & F; Fig. 3B & C), whereas GM1 is limited to myelinated fibers found in lower layers of cerebral cortex (Fig. 2C).

#### Basal ganglia

Both striatum (caudoputamen) and globus pallidus show strong immunoreactivity to gangliosides GD1a (Fig. 3B’), GD1b (data not shown) and GT1b (Fig. 3B’’), whereas GM1 is absent from these structures and only found in the neighboring white matter tracts (data not shown).

#### Amygdala

All amygdaloid nuclei show strong immunoreactivity to gangliosides GD1a (Figs. 2E, 3B’), GD1b (Fig. 2B) and GT1b (Figs. 2F, 3B’’), but are devoid of GM1 (Fig. 2C).

#### Hippocampal formation

Major brain gangliosides are differentially distributed in the hippocampal formation. Anti-GM1 immunostaining is limited to alveus hippocampi and few other fibers found in oriens layer and lacunosum moleculare layer of hippocampus, as well as in polymorph layer of dentate gyrus. GM1 is absent from pyramidal cells of the CA1 and CA3 fields of Ammon’s horn (Fig. 3D2, 3E2) and granule cells of dentate gyrus (Fig. 3F2). All layers of the hippocampus and dentate gyrus are positive for gangliosides GD1a, GD1b and GT1b. GD1a is strongly expressed in pyramidal cells of the CA3 field of Ammon’s horn (Fig. 3E3) and granule cells of dentate gyrus (Fig. 3F3). Moderate expression of gangliosides GD1a (Fig. 3D3) and GT1b (Fig. 3D5) is found in pyramidal cells of CA1 field. Granule cells of dentate gyrus also show moderate expression of GD1b (Fig. 3F4) and GT1b (Fig. 3F5). Weak expression of GD1b is found in pyramidal cells of CA1 (Fig. 3D4) and CA3 (Fig. 3E4) field, with CA3 field being also weakly positive for GT1b (Fig. 3E5). The pattern of expression of gangliosides GD1a, GD1b and GT1b is consistent with expression primarily on neuronal plasma membranes.

#### White matter tracts of the telencephalon

**Figure 4:**
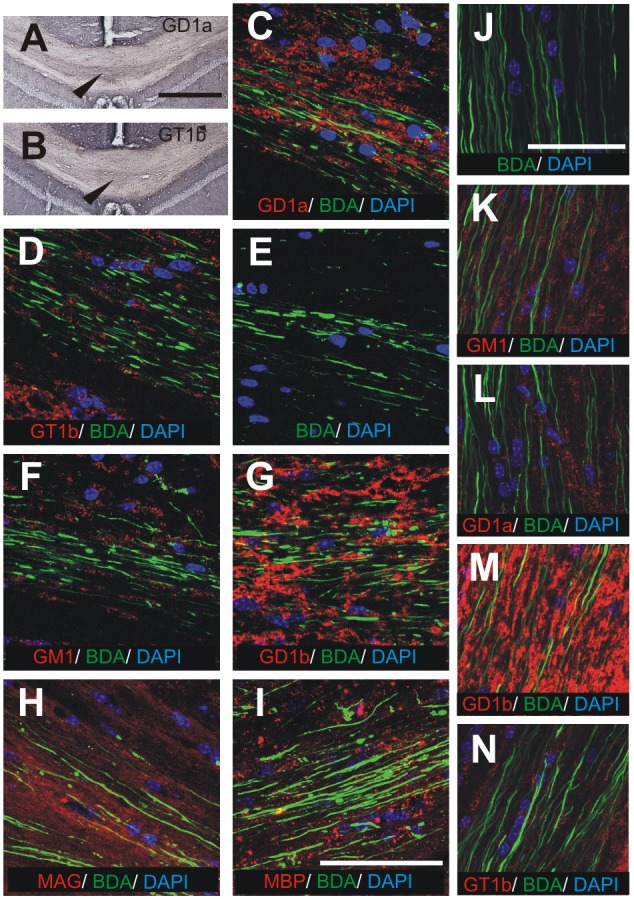
The expression of gangliosides GM1 (F, K; red), GD1a (A, C, L; red), GD1b (G, M; red), GT1b (B, D, N; red), MAG (H, red) and MBP (I, red) in coronal sections of corpus callosum at the level of habenular nuclei (A-I) and horizontal sections of corticospinal tract in cervical spinal cord (J-N). Commissural fibers and corticospinal tract are labeled with BDA (C-N; green). A subset of GD1a and GT1b expressing fibers are detected in the middle of corpus callosum (A, B; arrowheads). Cell nuclei are stained with DAPI (blue). The negative control is performed by omitting the primary antibody (E, J). Scale bars = 500 µm in A, B and 50 µm in C-N.

Gangliosides GD1a and GT1b are expressed in some white matter tracts of the telencephalon, but not others. Consistent with their expression on myelinated axons, gangliosides GD1a and GT1b immunodetection was found to be satisfactory only if tracts were cut longitudinally instead of perpendicularly. For that reason, brains cut in three planes (coronal, sagittal and horizontal) were analyzed. A subset of fibers in the corpus callosum show high immunoreactivity for GD1a (Figs. 4A, C) and GT1b (Figs. 4B, D). Based on anterograde BDA tracing (from the sensorimotor cortex of right hemisphere) GD1a- and GT1b-labeled fibers were identified as myelinated commissural fibers, as shown on consecutive sections double-stained with Alexa Fluor 488 conjugated streptavidin (for detection of BDA) and anti-MAG (Fig. 4H) or anti-MBP (Fig. 4I) antibodies. In contrast, gangliosides GM1 (Fig. 4F) and GD1b (Fig. 4G) are expressed throughout corpus callosum.

### Diencephalon

#### Thalamus

Most thalamic nuclei abundantly express gangliosides GM1 (Fig. 2C), GD1a (Figs. 2E, 3B’, 3C’), GD1b (Fig. 2B) and GT1b (Figs. 2F, 3B’’, 3C’’). However, GD1a is only weakly expressed in reticular nucleus of thalamus (Fig. 3B’).

#### Epithalamus

Medial and lateral habenular nuclei show strong expression of GD1b (data not shown) and GT1b (Fig. 3C’’, asterix). While there is a weak expression of GM1 (data not shown), GD1a is completely absent from these structures (Fig. 3C’).

#### Hypothalamus

All hypothalamic nuclei, including paraventricular and medial preoptic nuclei and anterior hypothalamic area, abundantly express gangliosides GM1 (Fig. 2C), GD1a (Figs. 2E, 3B’, 3C’), GD1b (Fig. 2B) and GT1b (Figs. 2F, 3B’’, 3C’’).

#### Subthalamus

While GT1b is expressed both in subthalamic nucleus and zona incerta (Fig. 3G’’), GD1a is completely absent from these structures (Fig. 3G’).

### Cerebellum

**Figure 5:**
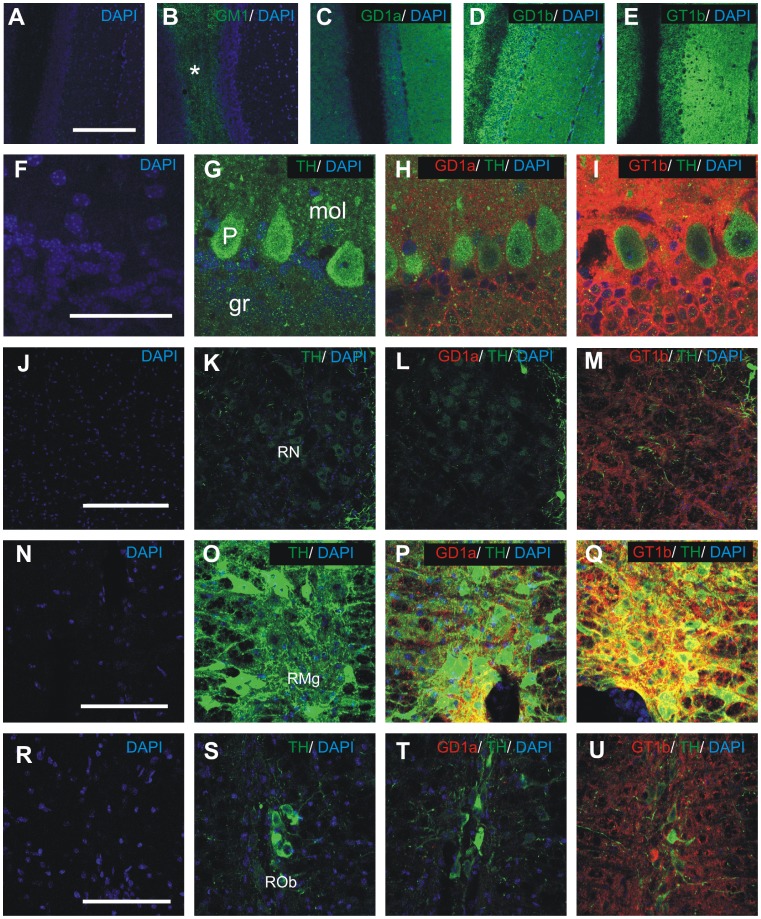
The expression of major brain gangliosides in coronal sections of mouse cerebellum (A-I), red nucleus (J-M) and raphe nuclei (N-U). Distribution of gangliosides GM1 (B, green, asterix denotes the white matter of cerebellum), GD1a (C, green), GD1b (D, green) and GT1b (E, green) in mouse cerebellum (low magnification). Double immunohistochemistry on GD1a (H, L, P, T; red) or GT1b (I, M, Q, U; red) and TH (G, H, I, K, L, M, O, P, Q, S, T, U; green) in cerebellum (F-I), red nucleus (J-M), raphe magnus nucleus (N-Q) and raphe obscurus nucleus (R-U). The negative controls were performed by omitting the anti-ganglioside antibody (A, G, K, O, S) or both anti-ganglioside and anti-TH antibody (F, J, N, R). Cell nuclei are stained blue using DAPI. gr, granular cell layer; mol, molecular layer; P, Purkinje cell layer; RMg, raphe magnus; RN, red nucleus; ROb, raphe obscurus. Scale bars = 200 µm in A-E, J-M; 50 µm in F-I and 100 µm in N-U.

In the mouse cerebellum, GM1 is detected only in white matter (Fig. 5B, asterix), while gangliosides GD1a (Figs. 5C, H), GD1b (Fig. 5D) and GT1b (Fig. 5E, I) are found in all three layers of cerebellar cortex (granule cell layer, Purkinje cell layer and molecular layer). It is not clear, however, if gangliosides GD1a and GT1b are expressed in cellular membranes of Purkinje cells or in the synapses and fibers surrounding them. The white matter of cerebellum and cerebellar nuclei show strong immunoreactivity for GM1 (Fig. 2C) and GD1b (Fig. 2B) and a slight immunoreactivity for GT1b (Fig. 2F), while GD1a is completely absent from these structures (Fig. 2E).

### Brainstem

Gangliosides GM1, GD1b and GT1b are present in most nuclei of the brainstem and in white matter tracts. In midbrain, moderate expression of GT1b is found both in reticular and compact part of substantia nigra (Fig. 3G’’) and red nucleus (Fig. 5M). While GD1a is strongly expressed in both parts of substantia nigra (Fig 3G’), no immunoreactivity is found in the red nucleus (Fig. 5L).

The raphe magnus and pallidus nuclei show strong expression of GT1b (Fig. 5Q) and moderate expression of GD1a (Fig. 5P), whereas the raphe obscurus nucleus shows only moderate expression of GT1b (Fig. 5U) and no GD1a (Fig. 5T).

**Figure 6:**
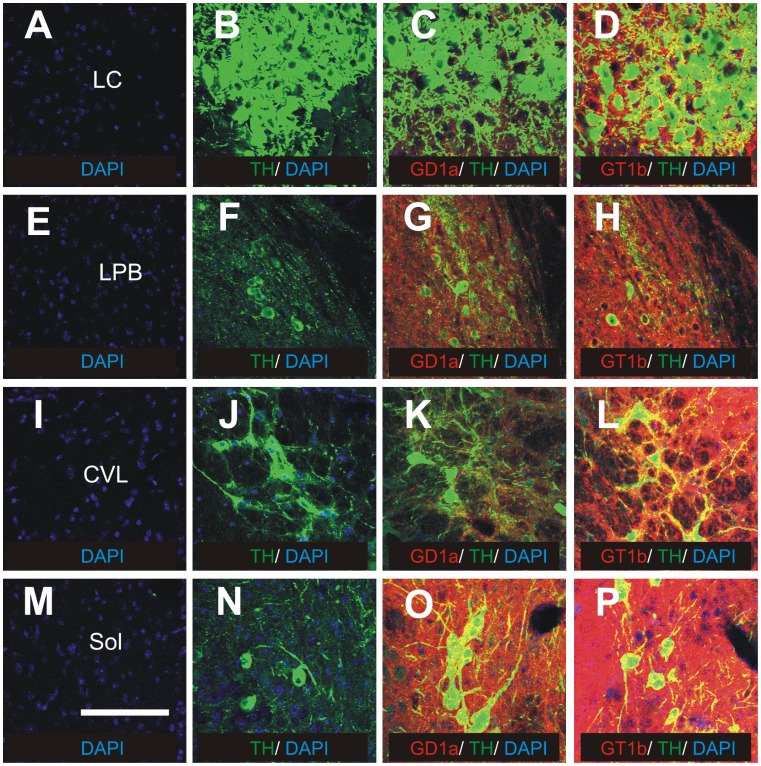
The distribution of gangliosides GD1a and GT1b in autonomic nuclei of brainstem (coronal sections). Double immunohistochemistry with anti-tyrosine hydroxylase (TH) antibody (B-D, F-H, J-L, N-P, green) and anti-GD1a (C, G, K, O; red) or anti-GT1b (D, H, L, P; red). Locus coeruleus (A-D), lateral parabrachial nucleus (E-H), caudal ventrolateral medulla (I-L) and solitary nucleus (M-P). The negative controls (A, E, I, M) were performed by omitting primary antibodies. Cell nuclei are stained blue with DAPI. CVL, caudal ventrolateral medulla; LC, locus coeruleus; LPB, lateral parabrachial nucleus; Sol, solitary nucleus. Scale bar = 100 µm.

**Figure 7:**
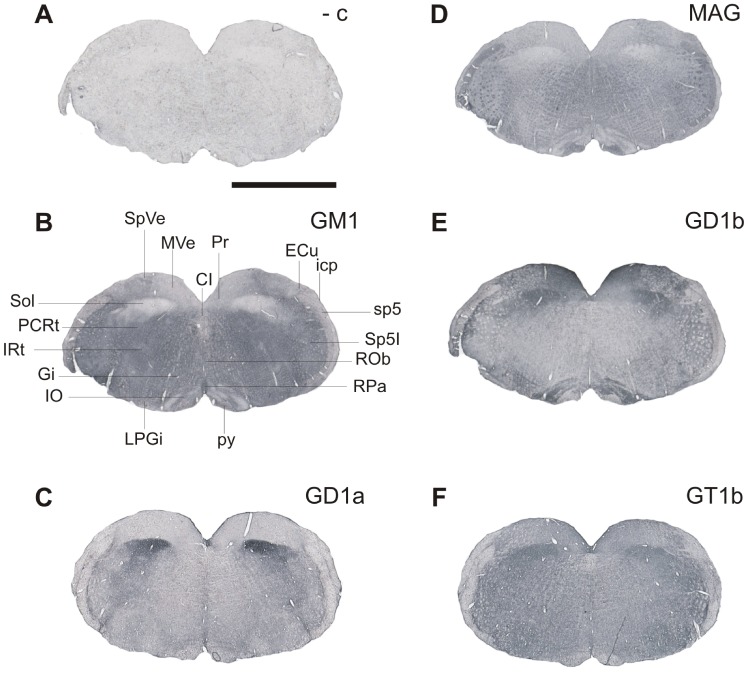
The distribution of gangliosides in the nuclei of brainstem at the level of inferior olives: GM1 (B), GD1a (C), GD1b (E) and GT1b (F). MAG is shown for comparison and is expressed in myelinated fibers (D). The negative control was performed by omitting the primary antibody (A). CI, caudal interstitial nucleus of the medial longitudinal fasciculus; ECu, external cuneate nucleus; Gi, gigantocellular reticular nucleus; icp, inferior cerebellar peduncule; IO, inferior olive; IRt, intermediate reticular nucleus; LPGi, lateral paragigantocellular nucleus; MVe, medial vestibular nucleus; PCRt, parvicellular reticular nucleus; Pr, prepositus nucleus; py, pyramidal tract; ROb, raphe obscurus nucleus; RPa, raphe pallidus nucleus; Sol, solitary nucleus; SpVe, spinal vestibular nucleus; sp5, spinal trigeminal tract; Sp5I – spinal trigeminal nucleus. Scale bar = 2000 µm.

Among autonomic nuclei of the brainstem, strong GD1a immunoreactivity is detected in periaqueductal gray matter, locus coeruleus (Fig. 6C), subcoeruleus nucleus, medial parabrachial nucleus, lateral parabrachial nucleus (Fig. 6G), Kolliker-Fuse nucleus, rostral ventrolateral medulla, caudal ventrolateral medulla (Fig. 6K) and solitary nucleus (Fig. 6O). Strong expression of GT1b is found in locus coeruleus (Fig. 6D), lateral parabrachial nucleus (Fig. 6H), caudal ventrolateral medulla (Fig. 6L), solitary nucleus (Fig. 6P) and other brainstem nuclei that are also positive for GD1a. GM1 and GD1b are moderately expressed in all of these autonomic nuclei, except solitary nucleus which is devoid of GM1 (Fig. 7B).

Moderate expression of GD1a and GT1b is also found in mesencephalic, principal and spinal trigeminal nuclei, as well as in pontine, medullary, parvicellular and intermediate reticular nuclei (Fig. 7C, F), all of which project their axons to the spinal cord. The gigantocellular reticular nucleus, involved in control of blood pressure and axial musculature was also found to express GT1b (Fig. 7F) and GD1a, although GD1a expression is relatively weak (Fig. 7C). Interestingly, GD1a is not expressed in either of the vestibular nuclei (Fig. 7C).

GD1b is strongly expressed in inferior olivary, vestibular nuclei and solitary nucleus and to some extent in gigantocellular reticular nucleus, parvicellular reticular nucleus and intermediate reticular nucleus (Fig. 7E). The pattern of expression of GT1b is similar to that of GM1, the difference being only in the solitary nucleus that is positive for GT1b. It is also worth noticing that certain white matter tracts are positive for GT1b such as: spinal trigeminal tract, inferior cerebellar peduncule and pyramidal tract, the latter being also positive for GD1a (Figs. 7F and 7C, respectively).

### Spinal Cord

**Figure 8:**
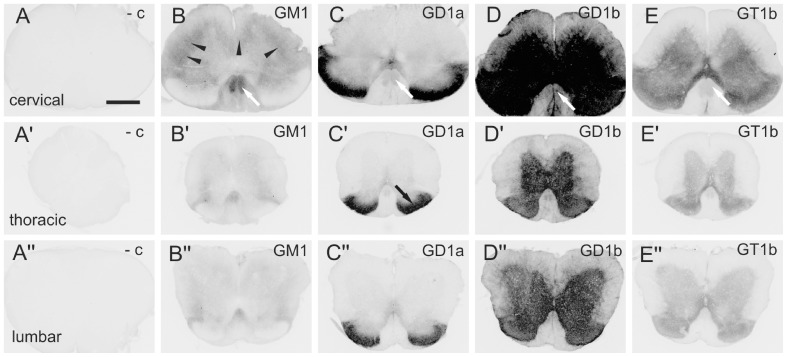
The distribution of gangliosides GM1(B, B’,B’’), GD1a (C, C’,C’’), GD1b (D, D’,D’’) and GT1b (E, E’,E’’) in transverse sections of mouse cervical (A, B, C, D, E), thoracic (A', B', C', D', E') and lumbar (A'', B'', C'', D'', E'') spinal cord. The negative control is performed by omitting the primary antibody (A, A’, A’’). Black arrowheads denote the propriospinal white matter tracts. White arrows point to the corticospinal tract. Black arrow points to the Rexed laminae I and II. Scale bar = 500 µm.

Whereas GD1b is expressed in both white and gray matter of the spinal cord (Figs. 8D, D’, D’’), other gangliosides are limited to more specific areas. GM1 is strongly expressed in corticospinal tract (Figs. 8B, B’, B’’; white arrow) and moderately expressed in other white matter, mainly in propriospinal tracts surrounding the gray matter (Figs. 8B, B’, B’’; black arrowheads). GM1 is also weakly expressed in gray matter. The expression of GD1a is strong in Rexed laminae 1 and 2 of dorsal horn, moderate around central canal and weak in other Rexed laminae of gray matter (Figs. 8C, C’,C’’). GT1b is moderately expressed in all Rexed laminae of gray matter (Fig. 8E, E’, E’’). Since corticospinal tract is the main pathway involved in skilled voluntary movements, such as food pellet reaching, the expression of gangliosides in horizontal sections of spinal cord has been studied in detail, namely by tracing the corticospinal tract with BDA tracer. Co-localization studies have revealed a strong signal for GD1b (Fig. 4M; red) and moderate signals for GM1 (Fig. 4K; red), GD1a (Fig. 4L; red) and GT1b (Fig. 4N; red) in corticospinal tract traced with BDA (green).

### Discussion

In this study, we report a detailed expression analysis of major brain gangliosides (GM1, GD1a, GD1b and GT1b) in the adult mouse CNS. Previously, the distribution of major brain gangliosides was studied by using immunohistochemistry and biochemistry [5]–[13].

Although gangliosides are major cell surface structures on all neurons, their immunohistochemical analysis is technically complicated by their lipid nature. Fixation of tissues with organic solvents [19] or exposure to detergents [20], [21] results in ganglioside extraction and/or redistribution [8], [9]. Since the same major ganglioside structures are shared by mammals and birds, generating high affinity and specific antibodies for each has also been a challenge [6], [9], [10]. Wild type mice, for example, do not typically mount a robust IgG response to gangliosides [11], and the isotype of the antibody affects immunodetection [11], [22].

To help mitigate these issues we used highly specific IgG-class monoclonal antibodies produced in *B4galnt1* null mice, which lack complex brain gangliosides and therefore mount a robust IgG response to major brain gangliosides [11], [18]. Immunostaining conditions were used that optimize ganglioside tissue retention and limit or eliminate ganglioside redistribution. Although the data are instructive, ganglioside immunoreactivity still depends on several factors: (a) the density of a particular ganglioside in the plasma membrane [23], (b) other components of the cell membrane [24] and (c) the ceramide portion of the ganglioside [25]. Therefore, the lack of immunoreactivity to a ganglioside epitope does not prove that the ganglioside is absent. However, by using well matched and specific IgG-class anti-ganglioside antibodies under well characterized conditions, reasonable comparisons can be inferred with respect to antibody-accessible structures.

A complementary method for analysis of ganglioside distribution in CNS tissue is imaging mass spectrometry (IMS). IMS gives detailed information on the ceramide core of the ganglioside and is able to assess multiple ganglioside species at a time in a single tissue slice. However, it has the well documented potential problem of partial loss of sialic acid during ionization and differentiation between ganglioside isomers (such as GD1a and GD1b) requires more detailed (MS^n^) analyses [15]–[17].

Our results show that GD1b and GT1b are widely expressed throughout the mouse CNS, whereas the immunoreactivity to GM1 is predominately in white matter tracts and immunoreactivity to GD1a significantly decreases caudal to the superior colliculus. Particularly strong expression of GD1a, GD1b and GT1b is found in olfactory bulbs, neocortex, basal ganglia, amygdaloid nuclear complex, septal regions, thalamic nuclei, hypothalamic nuclei and hippocampal formation.

Strong expression of GD1b and GT1b is found in all three layers of cerebellum (molecular layer, Purkinje cell layer and granular cell layer) and cerebellar nuclei, whereas GD1a is moderately expressed in cerebellar cortex, but completely absent from cerebellar nuclei. Our results also show that GM1 is limited to white matter in the cerebellum.

Among nuclei of brainstem, strong immunoreactivity to GD1a is detected in periaqueductal gray matter, locus coeruleus, subcoeruleus nucleus, medial and lateral parabrachial nucleus, Kolliker-Fuse nucleus, rostral and caudal ventrolateral medulla and solitary nucleus. Interestingly, those are all autonomic nuclei that are also positive for tyrosine hydroxylase. It is important to note that these nuclei send their projections to the spinal cord and that they are involved in cardiovascular reflexes and respiratory control but also receive sensory, nociceptive and visceral input from periphery. In concordance with these results, previous biochemical studies have also shown the expression of GD1a and GT1b in locus coeruleus, as well as in dorsal raphe nucleus, laterodorsal tegmentum and pedunculopontine tegmentum [5].

On the other hand, immunoreactivity to GD1a is weak or absent in the reticular nucleus of thalamus, habenular nuclei, zona incerta, subthalamic nucleus, red nucleus, superior and inferior colliculus, cochlear nuclei, vestibular nuclei, dorsal tegmental nucleus, motor nuclei of the trigeminal nerve, facial nucleus, hypoglossal nucleus, gracile nucleus, cuneate nucleus, external cuneate nucleus and ventral horn of spinal cord (for detailed comparison of GD1a and GT1b expression see Table S2).

Another interesting finding is that certain white matter tracts such as corpus callosum, the main comissural pathway of telencephalon, and corticospinal tract express all four major gangliosides, while others, e.g. white matter of cerebellum, do not. It is not clear from our data if certain gangliosides are on axolemma or on myelin membrane. However, biochemical studies have shown that GM1 is part of myelin membrane, while CNS axolemma contains all four major brain gangliosides [26].

A goal in documenting the distribution of major brain gangliosides is to inform theories about their functions. Based on studies of the *B4galnt1*-null mouse, in which all complex gangliosides are replaced by the simpler structures GM3 and GD3, major brain gangliosides have functional roles in long-term axon-myelin stabilization [27]. Other studies implicate GD1a and GT1b as complementary receptors for myelin-associated glycoprotein (MAG), a ganglioside-binding protein expressed selectively on myelin membranes apposed to the axon surface. Mice engineered to lack MAG, complex gangliosides, or double null mice have similar long-term dysmyelination phenotypes [28]. Together, these data implicate GD1a and/or GT1b as receptors in axon-myelin interactions.

MAG is also known to inhibit axon outgrowth *in vitro*, and has been proposed as one of the axon regeneration inhibitors that limits recovery after CNS injury. MAG has several receptors on nerve cells that mediate axon outgrowth inhibition, including gangliosides [29], Nogo receptors (NgR’s) [30], β-integrin [31] and PirB [32]. Comparing the distribution of MAG receptors GD1a/GT1b to other MAG receptors may contribute to our understanding of selective MAG functions and mechanisms in different neuronal pathways. In this light, it is interesting to note the distribution of NgR’s in the CNS in comparison to gangliosides GD1a and GT1b. Most of the studies on NgR distribution have used *in situ* hybridization, although immunohistochemistry on NgR protein has been reported by several groups [33], [34]. All studies agree that NgR’s are expressed in neocortical neurons, pyramidal cells of hippocampus, granule cells of dentate gyrus, most of thalamic nuclei and granule cells of cerebellum.

Two recent *in vitro* studies revealed that different neurons respond to MAG inhibition of neurite outgrowth using different signaling pathways [35], [36]. While, cerebellar granule neurons (CGNs) respond to MAG inhibition by a ganglioside – dependent pathway, most of MAG-mediated neurite outgrowth inhibition in DRGs is mainly through NgR-dependent pathway. In addition, postnatal hippocampal neurons and retinal ganglion cells use both pathways for MAG-mediated inhibition. In the light of expression of NgR1-3 and gangliosides GD1a and GT1b in these cells in the adult mouse CNS, it is expected that hippocampal neurons, which strongly express both NgR1-3 [33], [34] and gangliosides GD1a and GT1b, respond to MAG inhibition using both receptors. Interestingly, while CGNs express both gangliosides GD1a and GT1b and NgRs, they respond to MAG inhibition primarily through ganglioside- dependent pathway. The explanation for this could be in the lack of NgR co-receptors or other downstream signaling molecules in CGNs.

The studies of the distribution of major brain gangliosides in DRGs have shown that most neurons express GT1b, while GD1a is expressed mainly in small neurons (15–30 µm diameter), involved in nociception, and only in 10–20% of larger neurons [7]. This may explain why most of the MAG-mediated inhibition in DRG cultures is NgR-dependent.

It is important to note that gangliosides GD1a and GT1b are strongly expressed in cerebral cortex, corticospinal tract and certain brainstem nuclei that comprise ascending and descending tracts of spinal cord. However GD1a and GT1b expression is weak in gracile and cuneate nuclei that comprise dorsal column pathway. This knowledge may be helpful in designing treatments that address MAG inhibition of axonal regeneration through manipulation of these particular gangliosides. Along the same line, pathway-specific treatments may be designed based on the specific MAG receptor expression profiles. This notion is supported by the findings that treatments that target a single molecule often fail to evoke robust axonal regeneration after spinal cord injury [37]–[39].

## Supporting Information

Table S1 Primary antibodies used in the study.
(DOCX)

Table S2 Qualitative analysis of immunohistochemical reactivity to gangliosides GD1a and GT1b. +++, strong signal; ++, moderate signal; +, weak signal, −, no signal.
(DOCX)
