# Supplementary material for: Differential Distribution of Major Brain Gangliosides in the Adult Mouse Central Nervous System
Source: PLoS One. 2013 Sep 30;8(9):e75720. doi: 10.1371/journal.pone.0075720 (PMC3787110; doi:10.1371/journal.pone.0075720)
Supplement: Table S1 — Primary antibodies used in the study. (DOCX) [file pone.0075720.s001.docx]

Table S1.

| **Antigen** | **Host and type** | **Immunogen** | **Source and Catalog No.** | **Concentration/ Dilution** |
| --- | --- | --- | --- | --- |
| GM1 | Mouse monoclonal | keyhole limpet hemocyanin conjugated bovine brain GM1 | Schnaar lab | 0.35 – 1.2 µg/ml |
| GD1a | Mouse monoclonal | keyhole limpet hemocyanin conjugated bovine brain GD1a | Schnaar lab | 0.35 – 1.2 µg/ml |
| GD1b | Mouse monoclonal | keyhole limpet hemocyanin conjugated bovine brain GD1b | Schnaar lab | 0.35 – 1.2 µg/ml |
| GT1b | Mouse monoclonal | keyhole limpet hemocyanin conjugated bovine brain GT1b | Schnaar lab | 0.35 – 1.2 µg/ml |
| MAG | Mouse monoclonal | L2 epitope carrying glycoprotein extract of 1-2 day old chicken brain | Millipore, MAB1567, clone 513 | 1.2 µg/ml |
| MBP | Mouse monoclonal | Purified human myelin basic protein | QED Biosciences, 24201 | 1.5 µg/ml |
| TH | Rabbit polyclonal | Denatured tyrosine hydroxylase from rat pheochromocytoma (denatured by sodium dodecyl sulfate) | Millipore, AB152 | 1:1000 |
