# Supplementary material for: Differential Distribution of Major Brain Gangliosides in the Adult Mouse Central Nervous System
Source: PLoS One. 2013 Sep 30;8(9):e75720. doi: 10.1371/journal.pone.0075720 (PMC3787110; doi:10.1371/journal.pone.0075720)
Supplement: Table S2 — Qualitative analysis of immunohistochemical reactivity to gangliosides GD1a and GT1b. +++, strong signal; ++, moderate signal; +, weak signal, −, no signal. (DOCX) [file pone.0075720.s002.docx]

Table S2.

| **Brain area** | **GD1a** | **GT1b** |
| --- | --- | --- |
| 1. telencephalon |  |  |
| olfactory bulb: |  |  |
| glomerular layer | +++ | + |
| mitral layer | +++ | +++ |
| granular layer | +++ | +++ |
| accessory olfactory bulb | +++ | -/+ |
| piriform cortex | +++ | +++ |
| striatum (caudoputamen) | +++ | +++ |
| globus pallidus | +++ | +++ |
| septal nuclei | ++ | ++ |
| amygdaloid nuclear complex |  |  |
| corticomedial nuclear group | +++ | +++ |
| basolateral nuclear group | +++ | +++ |
| stria terminalis | + | + |
| hippocampal formation |  |  |
| Ammon's horn, field CA1 | ++ | ++ |
| Ammon's horn, field CA3 | +++ | + |
| dentate gyrus, granular layer | +++ | ++ |
| fornix | - | + |
| neocortex | +++ | +++ |
| corpus callosum | + | + |
|  |  |  |
| 1. diencephalon |  |  |
| habenular nuclei | - | ++ |
| thalamus |  |  |
| reticular nucleus | + | +++ |
| other nuclei | +++ | +++ |
| hypothalamus |  |  |
| lateral hypothalamic area | +++ | +++ |
| supraoptic nucleus | +++ | +++ |
| paraventricular nucleus |  |  |
| posterior hypothalamic area | +++ | +++ |
| other nuclei | +++ | +++ |
| zona incerta | - | +++ |
| subthalamic nucleus | - | +++ |
|  |  |  |
| 1. mesencephalon |  |  |
| periaqueductal gray | +++ | +++ |
| substantia nigra | +++ | ++ |
| ventral tegmental area | + | + |
| oculomotor nuclear complex | ++ | ++ |
| accessory oculomotor nuclei | ++ | ++ |
| visual system |  |  |
| pretectal area | + | +++ |
| superior colliculus | + | +++ |
| red nucleus | - | ++ |
| auditory system |  |  |
| inferior colliculus | + | ++ |
| cochlear nucleus | - | ++ |
|  |  |  |
| 1. pons |  |  |
| pontine nuclei | ++ | ++ |
| laterodorsal tegmental nucleus | ++ | ++ |
| dorsal tegmental nucleus | - | ++ |
| parabrachial nuclei | +++ | +++ |
| locus coeruleus | +++ | +++ |
| subcoeruleus nucleus | +++ | +++ |
|  |  |  |
| 1. cerebellum |  |  |
| cerebellar cortex |  |  |
| granular layer | ++ | +++ |
| Purkinje cell layer | + | + |
| molecular layer | ++ | +++ |
| cerebellar nuclei | - | ++ |
|  |  |  |
| 1. medulla oblongata |  |  |
| raphe nuclei |  |  |
| dorsal raphe | ++ | ++ |
| raphe magnus | ++ | +++ |
| raphe obscurus | - | ++ |
| trigeminal nuclei |  |  |
| mesencephalic trigeminal nucleus | ++ | ++ |
| principal sensory nucleus | ++ | ++ |
| motor nuclei of the trigeminal nerve | - | ++ |
| vestibular nuclei | - | ++ |
| reticular nuclei |  |  |
| gigantocellular nuclear complex | + | ++ |
| ambiguus nuclei | ++ | ++ |
| lateral reticular nucleus | ++ | ++ |
| facial nucleus | - | + |
| nucleus of the solitary tract | +++ | +++ |
| caudal ventrolateral medulla | +++ | +++ |
| external cuneate nucleus | - | + |
| inferior olivary complex | + | ++ |
| hypoglossal nucleus | - | ++ |
| dorsal nucleus of the vagus nerve | ++ | ++ |
| gracile nucleus | - | + |
| cuneate nucleus | - | + |
|  |  |  |
| 1. spinal cord |  |  |
| gray matter |  |  |
| dorsal horn | +++ | ++ |
| ventral horn | -/+ | ++ |
| white matter (corticospinal tract) | + | + |
